# Feature Subset Selection with Optimal Adaptive Neuro-Fuzzy Systems for Bioinformatics Gene Expression Classification

**DOI:** 10.1155/2022/1698137

**Published:** 2022-05-14

**Authors:** Anwer Mustafa Hilal, Areej A. Malibari, Marwa Obayya, Jaber S. Alzahrani, Mohammad Alamgeer, Abdullah Mohamed, Abdelwahed Motwakel, Ishfaq Yaseen, Manar Ahmed Hamza, Abu Sarwar Zamani

**Affiliations:** ^1^Department of Computer and Self Development, Preparatory Year Deanship, Prince Sattam Bin Abdulaziz University, AlKharj, Saudi Arabia; ^2^Department of Industrial and Systems Engineering, College of Engineering, Princess Nourah Bint Abdulrahman University, P.O. Box 84428, Riyadh 11671, Saudi Arabia; ^3^Department of Biomedical Engineering, College of Engineering, Princess Nourah Bint Abdulrahman University, P.O.Box 84428, Riyadh 11671, Saudi Arabia; ^4^Department of Industrial Engineering, College of Engineering Alqunfudah, Umm Al-Qura University, Mecca, Saudi Arabia; ^5^Department of Information Systems, College of Science & Art Mahayil, King Khalid University, Abha, Saudi Arabia; ^6^Research Centre, Future University, Egypt, New Cairo 11845, Egypt

## Abstract

Recently, bioinformatics and computational biology-enabled applications such as gene expression analysis, cellular restoration, medical image processing, protein structure examination, and medical data classification utilize fuzzy systems in offering effective solutions and decisions. The latest developments of fuzzy systems with artificial intelligence techniques enable to design the effective microarray gene expression classification models. In this aspect, this study introduces a novel feature subset selection with optimal adaptive neuro-fuzzy inference system (FSS-OANFIS) for gene expression classification. The major aim of the FSS-OANFIS model is to detect and classify the gene expression data. To accomplish this, the FSS-OANFIS model designs an improved grey wolf optimizer-based feature selection (IGWO-FS) model to derive an optimal subset of features. Besides, the OANFIS model is employed for gene classification and the parameter tuning of the ANFIS model is adjusted by the use of coyote optimization algorithm (COA). The application of IGWO-FS and COA techniques helps in accomplishing enhanced microarray gene expression classification outcomes. The experimental validation of the FSS-OANFIS model has been performed using Leukemia, Prostate, DLBCL Stanford, and Colon Cancer datasets. The proposed FSS-OANFIS model has resulted in a maximum classification accuracy of 89.47%.

## 1. Introduction

Microarray is an advanced technology that helps to recognize the pattern of gene expression of various genes at a time at the genomic level. It supports the researcher to investigate and analyze millions of genes in a single experiment [1]. It identifies many present diseases connected to each individual gene such as anaemia and cancer. Analysis of Gene Expression provides a method to recognize the gene that is differentially expressed [2], which is accountable to develop some diseases. Also, it shows the difference between normal and abnormal genes through a mathematical model [3, 4]. Many openly accessible datasets such as Array Express and Gene Expression Omnibus (GEO) make the task easier to identify gene patterns of rare diseases. Classification of gene expression data splits cancer samples from healthy samples that are utilized in response to treatment prediction. Due to the smaller amount of samples with a larger amount of features in the gene expression information, the standard ML method disappoints to implement better for cancer classification [5].

Recently, there has been tremendous growth in the medical field around the world. There are several computational approaches utilized in the bioinformatics field in the last few decades, for example, data mining and pattern recognition, to deal with higher-dimensional problems but still unsuccessful [6]. Thus, recently, machine learning (ML), a branch of artificial intelligence, has received considerable attention from researchers in gene expression and genomics [7]. Also, ML is a branch of data science; the main goal is to allow a model for training and learning to make decisions by itself in the future. Machine learning is widely classified into semisupervised, semi-unsupervised, supervised, and unsupervised learning [8]. For microarray data classification, the ML-based feature selection (FS) techniques such as gene selection techniques assist in selecting the essential gene [9]. Feature selection assists to preserve useful attributes. It is mainly utilized for the higher-dimensional data; simply, FS is a dimensionality reduction method. Feature selection significantly assists in the field that has relatively scarce and samples too many features, e.g., DNA Microarray and RNA sequencing [10]. This approach assists in better understanding of the feature space, preventing the scare of model overfitting, maximizing the model training time, handling the dimension, and maximizing the prediction accuracy. The results of FS are the optimum amount of features that are related to the provided class label that contributed to the prediction process.

This study introduces a novel feature subset selection with optimal adaptive neuro-fuzzy inference system (FSS-OANFIS) for gene expression classification. The FSS-OANFIS model designs an improved grey wolf optimizer-based feature selection (IGWO-FS) model to derive an optimal subset of features. Besides, the OANFIS model is employed for gene classification and the parameter tuning of the ANFIS model is adjusted by the use of coyote optimization algorithm (COA). The application of IGWO-FS and COA techniques helps in accomplishing enhanced microarray gene expression classification outcomes. For examining the enhanced outcomes of the FSS-OANFIS model, a comprehensive simulation analysis was performed on distinct datasets.

## 2. Related Works

In reference [11], a two-phase approach named as ML-integrated ensemble of feature selection (FS) technique is used, and then a survival study was presented. In a primary stage, it can be chosen the optimum amongst 7 ML approaches dependent upon classifier accuracy, utilizing the whole group of features (under this case miRNAs). In the secondary stage, dependent upon classifier accuracy values, the top feature in all the FS approaches is assumed for making an ensemble to offer more categorization of miRNAs. Ayyad et al. [12] presented a novel classifier approach to gene expression data. Both executions are assumed that improve the performance of KNN. An important idea is for utilizing robust neighbors in trained data with utilizing a novel weighting approach. The authors in reference [13] presented a recently developed classification named Forest DNN (fDNN) for integrating the DNN structure with a supervised forest feature detector. Utilizing this built-in feature detector, this technique is capable of learning sparse feature representation and feeding the representation to NN for mitigating the overfitting problem. Dwivedi [14] developed a structure of approaches dependent upon supervised ML with utilizing the ANN approach for gene classification.

Shukla [15] established a novel gene selection (GS) approach by integrating minimum redundancy maximum relevance (mRMR) and teaching learning-based optimization (TLBO) for accurate cancer prediction. Primarily, during the presented method, mRMR was executed for determining one of the discriminative genes in the original feature set. In SVM, mRMR was utilized as a fitness function (FF) under the presented technique for selecting relevant features that are used for estimating the prediction accuracy and classifying cancer correctly. In reference [16], a novel social network analysis-based GS method was presented. The presented approach contains 2 important objectives: relevance maximization and redundancy minimization of chosen genes. During this approach, on all iterations, a maximal community was chosen repetitively. Next amongst the present genes under this community, the suitable genes were chosen by utilizing the node centrality-based condition.

In reference [17], an ensemble DL approach was presented for reducing the dimensional features. Primarily, the reduction of dimensional with utilize of auto-encoder (AE) by utilizing several hidden layers have occurred and under the next step, a folded AE is also utilized for reducing the dimensional of identical original data. Eventually, both are combined and top feature is chosen on the fundamental of T-score value. Forestiero et al. [18] presented a multiagent technique to create a distributing approach to DNA microarray management. The group of agents, whereas all one signifying a Microarray (or chip), implement from the parallel a sequence of easy functions exploiting local data and organized virtual infrastructure was created at a global level. The word embedded method, capable of capturing the semantic context and signifying microarray with vector, was utilized for mapping the chip, thus permitting advanced agent functions.

## 3. The Proposed Model

**Figure 1:**
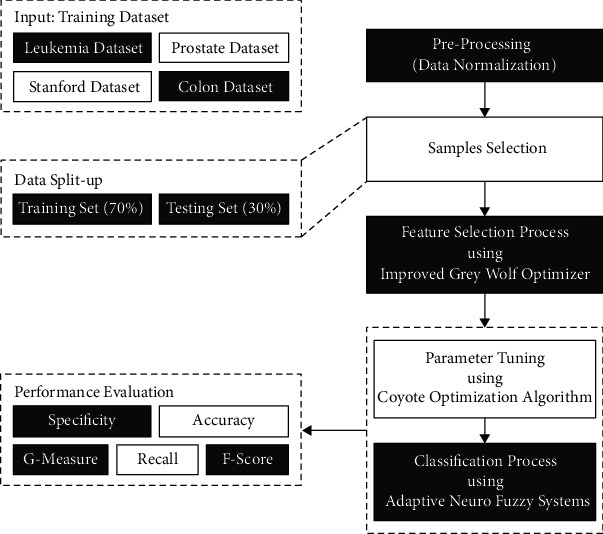
Overall process of FSS-OANFIS technique.

In this study, a new FSS-OANFIS model has been developed for microarray gene expression data classification. The presented FSS-OANFIS model encompasses a series of processes, namely, data preprocessing, IGWO-FS-based election of features, ANFIS classification, and COA-based parameter optimization. The application of IGWO-FS and COA techniques helps in accomplishing enhanced microarray gene expression classification outcomes.  Figure 1 shows the overall process of FSS-OANFIS technique.

### 3.1. Preprocessing

The z-score is a normalized and standardized system, which describes the count of standard deviation (SD), a raw data point, which is below or above the population mean [18]. It ideally lies in the range of −3 and +3. It standardizes the dataset to the aforementioned scale to change data with distinct scales to default scale. Thus, reflecting that several SD a point is below/above the mean as follows, but *x* refers to the value of particular instance, *μ* signifies the mean, and *σ* depicts the SD:

(1) $$\begin{array}{c} Z-\text{score}=\frac{\left(x-\mu \right)}{\sigma }. \end{array}$$

### 3.2. Steps Involved in IGWO-FS Technique

Once the input data is preprocessed, the next stage is to choose an optimal subset of features. The GWO algorithm is naturally inspired by the behavior and social leadership of the grey wolves [19]. The population of wolves can be classified into alpha, beta, delta, and omega for establishing the social hierarchy of wolves. The fittest solution is called alpha (*α*), whereas beta (*δ*) and delta (*δ*) represent the 2nd and 3rd most efficient options, respectively. Omega (*ω*) represents semblance of a hopeful solution. The arithmetical expression of readapting position 0 is shown as follows:

(2) $$\begin{array}{c} {\overset{⟶}{D}}_{\alpha }=\left|{\overset{⟶}{C}}_{1}\cdot {\overset{⟶}{X}}_{a}-\overset{⟶}{X}\right|,\\ {\overset{⟶}{D}}_{\beta }=\left|{\overset{⟶}{C}}_{2}\cdot {\overset{⟶}{X}}_{\beta }-\overset{⟶}{X}\right|,\\ {\overset{⟶}{D}}_{\delta }=\left|{\overset{⟶}{C}}_{3}\cdot {\overset{⟶}{X}}_{\delta }-\overset{⟶}{X}\right|, \end{array}$$

(3) $$\begin{array}{c} {\overset{⟶}{X}}_{1}={\overset{⟶}{X}}_{\alpha }-{\overset{⟶}{A}}_{1}\cdot \left({\overset{⟶}{D}}_{\alpha }\right),\\ {\overset{⟶}{X}}_{2}={\overset{⟶}{X}}_{\beta }-{\overset{⟶}{A}}_{2}\cdot \left({\overset{⟶}{D}}_{\beta }\right),\\ {\overset{⟶}{X}}_{3}={\overset{⟶}{X}}_{\delta }-{\overset{⟶}{A}}_{3}\cdot \left({\overset{⟶}{D}}_{\delta }\right), \end{array}$$

where ${\overset{⟶}{X}}_{a}$ denotes the location of the alpha, ${\overset{⟶}{X}}_{\beta }$ represent the location of the beta, ${\overset{⟶}{X}}_{\delta }$ indicates the location of the delta, and ${\overset{⟶}{C}}_{1},{\overset{⟶}{C}}_{2}$, and ${\overset{⟶}{C}}_{3}$ and ${\overset{⟶}{A}}_{1},{\overset{⟶}{A}}_{2}$, and ${\overset{⟶}{A}}_{3}$ signifies random vector, that is, the location of the existing solution, and shows the amount of iterations. It can be expressed in the following equation:

(4) $$\begin{array}{c} \overset{⟶}{T}\left(u+1\right)={\overset{⟶}{T}}_{p}\left(u\right)+\overset{⟶}{B}\cdot \overset{⟶}{E}, \end{array}$$

where $\overset{⟶}{E}$ is represented in equation (3), *u* indicates the iteration number, $\overset{⟶}{B},\overset{⟶}{D}$ denote the vector coefficient, and $\overset{⟶}{T_{p}}$ and $\overset{⟶}{T}$ represent the praise and grey wolf locations. The $\overset{⟶}{B},\overset{⟶}{D}$ vectors are calculated in the following equation:

(5) $$\begin{array}{c} \overset{⟶}{E}=\left|\overset{⟶}{D}\cdot {\overset{⟶}{T}}_{p}\left(u\right)-\overset{⟶}{T}\left(u\right)\right|,\\ \overset{⟶}{B}=2\overset{⟶}{b}\cdot \overset{⟶}{s_{1}}-\overset{⟶}{b},\\ \overset{⟶}{D}=2\overset{⟶}{s_{2}}. \end{array}$$

*s*_1_ and *s*_2_ denote vectors with arbitrary numbers within [0,1] and $\overset{⟶}{b}$ parameter is linearly reduced from 2 to 0 all over the iteration. Usually, the alpha is responsible for the chase. To change the position with the optimal searching agent position, the first three optimal solutions attained up until now compel another searching agent. Then, the wolves position can be upgraded as follows:

(6) $$\begin{array}{c} {\overset{⟶}{E}}_{\propto }=\left|{\overset{⟶}{D}}_{1}\cdot {\overset{⟶}{t}}_{\propto }-\overset{⟶}{Y}\right|,{\overset{⟶}{E}}_{\beta }=\left|{\overset{⟶}{D}}_{2}\cdot {\overset{⟶}{t}}_{\beta }-\overset{⟶}{Y}\right|,{\overset{⟶}{E}}_{\delta }=\left|{\overset{⟶}{D}}_{3}\cdot {\overset{⟶}{t}}_{\delta }\Rightarrow -\overset{⟶}{Y}\right|,\\ \overset{⟶}{t_{1}}=\left|\overset{⟶}{t_{\propto }}-\overset{⟶}{B_{1}}\cdot \overset{⟶}{E_{\propto }}\right|,\overset{⟶}{t_{2}}=\left|\overset{⟶}{t_{\beta }}-\overset{⟶}{B_{2}}\cdot \overset{⟶}{E_{\beta }}\right|,\overset{⟶}{t_{3}}=\left|\overset{⟶}{t_{\delta }}=\overset{⟶}{B_{3}}\cdot \overset{⟶}{E_{\delta }}\right|,\\ \overset{⟶}{t}\left(u+1\right)=\frac{\overset{⟶}{t_{1}}+\overset{⟶}{t_{2}}+\overset{⟶}{t_{3}}}{3}. \end{array}$$

The variable *b* governs the balance between exploration and exploitation. Here, the variable $\overset{⟶}{b}$ can be updated linearly in all the iterations, which ranges from 2 to 0, with *u* being the iteration number and *m*_*i*_ be the overall iterations allowed for the optimization:

(7) $$\begin{array}{c} \overset{⟶}{b}=2-u\cdot \frac{2}{m_{i}}. \end{array}$$

The wolf's location reflects attribute set selection and the solution space can be made by each probable attribute selection. The fitness function of the IGWO-FS technique can be utilized for determining whether an attribute subset would be selected or not:

(8) $$\begin{array}{c} \text{Fitness}=\propto^{*}\gamma_{S}\left(E\right)+\beta^{*}\frac{\left|D-S\right|}{\left|D\right|}. \end{array}$$

|*S*| represents the selected attribute length subset and *γ*_*S*_(*E*) denotes the classification quality of attribute set *S* in relation to decision *E*. The overall amount of quality indicates the letter |*D*|∝∈0,1 and *β*=1 − ∝, are two respective values for the attribute subset length and classification quality, repectively. Both have dissimilar implications for the attribute reduction task. The set ∝=0.9, *β*=0.1 and attribute subset length are less important when compared to the quality classification. The higher ensure that the ideal location is a rough set reduction as a minimum. The fitness function evaluates the quality of location. After defining the fitness level, important feature is taken as well as removing the unwanted feature.

The performance of the GWO algorithm can be improved by the design of IGWO algorithm with the inclusion of adaptive *β*‐hill climbing (A*β*HC). It is a recently presented local search-based technique, that is, basically, a modified version of *β*‐hill climbing (*βHC*) [20]. The study has established that A*β*HC gives optimum performance than several other famous local search techniques. For boosting the techniques exploitation capability and the quality of last solutions, *AβHC* has been combined with the fundamental GTO for support searching the neighborhoods of optimum solution under this study. The explanation of *AβHC* has been demonstrated mathematically as follows:

In order to provide an existing solution *X*_*i*_=(*x*_*i*,1_, *x*_*i*,2_,…, *x*_*i*,*D*_), *AβHC* is iteratively created an improved solution *X*_*i*_^″^=(*x*_*i*,1_^″^, *x*_*i*,2_^″^,…, *x*_*i*,*D*_^″^) on the fundamental of 2 control operators: *𝒩*− operator and *β*‐operator. The *N*‐operator primarily transfers *X*_*i*_ to a novel neighborhood solution.

*X*_*i*_′=(*x*_*i*,1_′, *x*_*i*,2_′,…, *x*_*i*,*D*_′) that is demonstrated in equations (9) and (10) as follows:

(9) $$\begin{array}{c} x_{ij}^{\prime }=x_{ij}\pm U\left(0,1\right)\times N,j=1,2,\ldots ,D, \end{array}$$

(10) $$\begin{array}{c} N\left(t\right)=1-\frac{t^{1/K}}{{\text{Maxiter}}^{1/K}}, \end{array}$$

where *U*(*O*, 1) refers the arbitrary number between the interval of 0 and 1 , *x*_*ij*_ represents the value of decision variable from the *j*^*th*^ dimensional, *t* stands for the existing iteration, Maxiter denotes the maximal count of iterations, *N* signifies the bandwidth distance amongst the existing solution and their neighbor, *D* refers to the spatial dimensionality, and the parameter *K* is a constant.

### 3.3. Optimal ANFIS-Based Classification

At the final stage, the OANFIS model has been employed for the detection and classification of gene expression data into multiple classes. A network with 2 inputs, *x* and *y* and one output, *f* is considered. The ANFIS is a fuzzy Sugeno method. For presenting the ANFIS structure, 2 fuzzy if-then rules dependent upon a first‐order Sugeno method are assumed as follows [21]:

- Rule 1: if*x* is *A*_1_ and *y* is *B*_1_, then *f*_1_=*p*_1_*x*+*q*_1_*y*+*r*_1_
- Rule 2: if *x* is *A*_2_ and *y* is *B*_2_, then *f*_2_=*p*_2_*x*+*q*_2_*y*+*r*_2_

**Figure 2:**
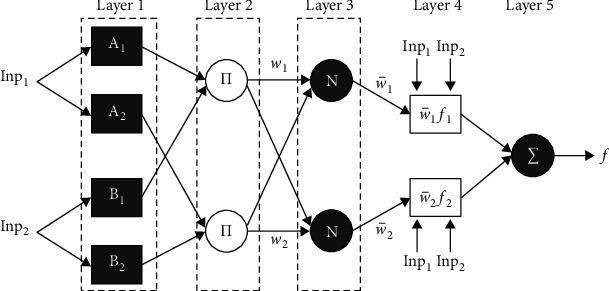
Structure of ANFIS.

Where *x* and *y* are inputs, *A*_1_ and *B*_*i*_ imply fuzzy sets, *f*_*i*_ is the output of fuzzy system, and *p*_*i*_, *q*_*i*_, and *r*_*i*_ represent the design parameters that are defined in the training procedure. The ANFIS structure for implementing these 2 rules, whereas a circle represents the set node and a square refers the adaptive node. The ANFIS infrastructure has 5 layers.  Figure 2 showss the framework of ANFIS.

*Layer 1*. All nodes from layer1 are adaptation nodes. The resultant of layer 1 is are fuzzy membership grade of the inputs that are provided as follows:

(11) $$\begin{array}{c} O_{i}^{1}=\mu_{A}\left(x\right)i=1,2,\\ O_{i}^{1}=\mu_{B_{i-2}}\left(x\right)i=3,4, \end{array}$$

where *x* and *y* refer the inputs to node *i*, *A* refers the linguistic label, and ∝_*A*_*i*__(*x*) and *μ*_*B*_*i*−2__(*x*) are some fuzzy membership functions. Generally, ∝_*A*_*i*__(*x*) is chosen as

(12) $$\begin{array}{c} \mu_{A_{i}}=\frac{1}{1+{\left\{{\left[\left(x-c_{i}\right)/a_{i}\right]}^{2}\right\}}^{b_{i}}}, \end{array}$$

where *a*_*i*_, *b*_*i*_, and *c*_*i*_ are the parameters of membership bell‐shaped function.

*Layer 2*. The node of this layer is labeled *M*, signifying that it can be implemented as an easy multiplier The resultant of this layer is demonstrated as follows:

(13) $$\begin{array}{c} O_{i}^{2}=w_{i}=\mu_{A_{i}}\left(x\right)\mu_{B_{i}}\left(y\right)i=1,2. \end{array}$$

*Layer 3*. It comprises set nodes which compute the ratio of firing strength of the rules as follows:

(14) $$\begin{array}{c} O_{i}^{3}={\bar{w}}_{i}=\frac{w_{i}}{w_{1}+w_{2}}i=1,2. \end{array}$$

*Layer 4*. During this layer, the adaptive node is used. The resultants of this layer are calculated by the following equation:

(15) $$\begin{array}{c} O_{i}^{4}={\bar{w}}_{i}f_{i}={\bar{w}}_{i}\left(p_{i}x+q_{i}y+r_{i}\right)i=1,2. \end{array}$$

${\bar{w}}_{i}$ signifies the normalized firing strength in layer 3.

*Layer 5*. The node executes the summation of every incoming signal. Therefore, an entire result of the model is provided as follows:

(16) $$\begin{array}{c} O_{i}^{5}=\sum_{i}{\bar{w}}_{i}f_{i}=\frac{\sum_{i}w_{i}f_{i}}{\sum_{i}w_{i}}. \end{array}$$

It could be realized that there are 2 adaptive layers under this ANFIS structure such as the 1st layer and 4th layer. During the 1st layer, there are 3 modifiable parameters {*a*, *b*_*i*_*c*_*i*_} that are connected to the input membership function. These parameters are usually named as premise parameters. During the 4th layer, there are also 3 modifiable parameters {*p*_*i*_*q*_*i*_*r*_*i*_} relating to the first‐order polynomial. This parameter is supposed the consequent parameter.

For tuning the ANFIS parameters, the COA is applied to it. The COA is a mathematical model that depends on smart diversity [22]. Chasing, driving, attacking, and blocking are archived by four distinct kinds of chimps that are attained by chasers, drivers, attackers, and obstacles. These hunting steps are accomplished in two phases such as exploration and exploitation stages. The exploration phase involves chasing, driving, and blocking the prey. The exploitation phase should attack the prey, and the chasing and driving are characterized as follows:

(17) $$\begin{array}{c} d=\left|c\cdot x_{\text{prey}}\left(t\right)-m\cdot x_{\text{chimp}}\left(t\right)\right|,\\ x_{\text{chimp}}\left(t+1\right)=x_{\text{prey}}\left(t\right)-a\cdot d, \end{array}$$

where *X*_prey_ denotes the vector of prey location, *x*_chimp_ indicates the vector of chimp location, *t* denotes the amount of present iterations, *a*, *c*, and *m* represent coefficient vectors and are calculated as follows:

(18) $$\begin{array}{c} a=2\cdot f\cdot r_{1}‐f,\\ c=2\cdot r_{2},\\ m=\text{chaotic}-\text{value}, \end{array}$$

where *f* declined nonlinearly from 2.5 to 0, *r*_1_ and *r*_2_ denote the random value within [0, 1], and *m* represents the chaotic vector. The dynamic coefficient *f* is selected for distinct slopes and curves; therefore, chimps employ distinct capabilities for searching the prey. Chimps upgrade the position according to the other chimps, and the arithmetical expression can be given in the following equation:

(19) $$\begin{array}{c} d_{\text{Attacker}}=\left|c_{1}x_{\text{Attacker}}-m_{1^{\chi }}\right|,\\ d_{\text{Barrier}}=\left|c_{2}x_{\text{Barrier}}-m_{2^{\chi }}\right|,\\ d_{\text{Chaser}}=|c_{3}x_{\text{Chaser}}-m_{3}x|\\ d_{\text{Driver}}=\left|c_{4}x_{\text{Driver}}-m_{4}x\right|,\\ x_{1}=x_{\text{Attacker}}-a_{1}\left(d_{\text{Attacker}}\right),\\ x_{2}=x_{\text{Barrier}}-a_{2}\left(d_{\text{Barrier}}\right),\\ x_{3}=x_{\text{Chaser}}-a_{3}\left(d_{\text{Chaser}}\right),\\ x_{4}=x_{\text{Driver}}-a_{4}\left(d_{\text{Driver}}\right),\\ x\left(t+1\right)=\frac{x+x_{2}+x_{3}+\chi_{4}}{4}. \end{array}$$

## 4. Experimental Validation

In this section, the experimental validation of the FSS-OANFIS model has been performed using four benchmark datasets [23–26]. The details of the dataset are given in Table 1. The results are investigated and the outcomes are assessed in terms of different measures. For experimental validation, a 10-fold cross-validation process is utilized.

### 4.1. Results Analysis of Proposed Model

**Figure 3:**
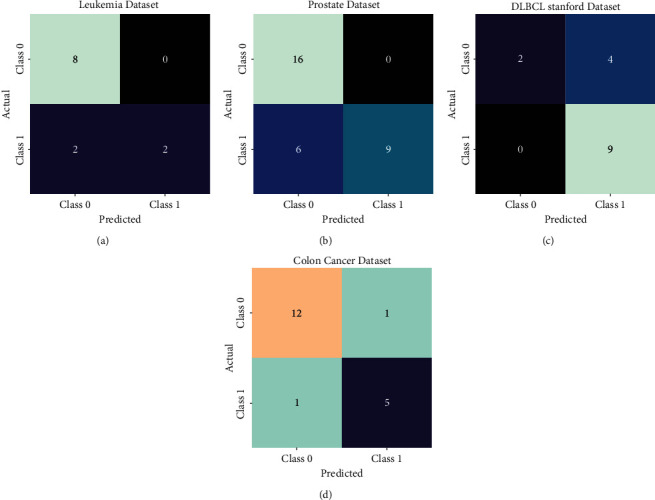
Confusion matrix of FSS-OANFIS technique under four datasets.

Figure 3 illustrates a set of confusion matrices offered by the FSS-OANFIS model on test datasets. The figure reported that the FSS-OANFIS model has properly recognized the class labels on all datasets. For instance, on the Leukemia dataset, the FSS-OANFIS model has identified 8 samples in class 0 and 2 samples in class 1. In addition, on the Prostate dataset, the FSS-OANFIS system has identified 16 samples in class 0 and 9 samples in class 1. Also, on the DLBCL Stanford dataset, the FSS-OANFIS approach has identified 2 samples in class 0 and 9 samples in class 1. Besides, on the Colon Cancer dataset, the FSS-OANFIS algorithm has identified 12 samples in class 0 and 5 samples in class 1.

Table 2 provides the overall classification outcomes of the FSS-OANFIS model on the test datasets. The experimental outcomes pointed out that the FSS-OANFIS model has offered effective outcomes on all the datasets applied. For instance, with the Leukemia dataset, the FSS-OANFIS model has resulted in average accu_*y*_, reca_*l*_, spec_*y*_, *F*_score_, and *G*_measure_ of 83.33%, 75%, 75%, 77.78%, and 80.08%, respectively. Following this, with the Prostate dataset, the FSS-OANFIS methodology has resulted in average accu_*y*_, reca_*l*_, spec_*y*_, *F*_score_, and *G*_measure_ of 80.65%, 80%, 80%, 79.61%, and 81.37%, respectively. Meanwhile, with the DLBCL Stanford dataset, the FSS-OANFIS algorithm has resulted in average accu_*y*_, reca_*l*_, spec_*y*_, *F*_score_, and *G*_measure_ of 73.33%, 66.67%, 66.67%, 65.91%, and 70.47%, respectively. Eventually, with the Colon Cancer dataset, the FSS-OANFIS technique has resulted in average accu_*y*_, reca_*l*_, spec_*y*_, *F*_score_, and *G*_measure_ of 89.47%, 87.82%, 87.82%, 87.82%, and 87.82%, respectively.

**Figure 4:**
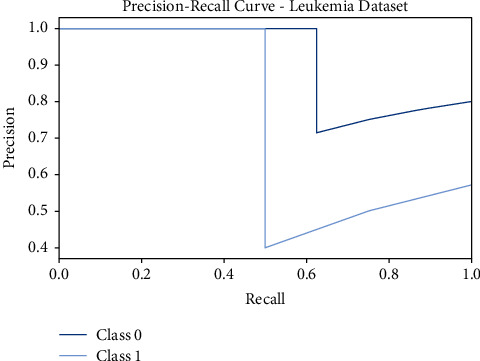
Precision-recall analysis of FSS-OANFIS technique under the Leukemia dataset.

Figure 4 shows the precision-recall curves offered by the FSS-OANFIS model on the test Leukemia dataset. The figure indicated that the FSS-OANFIS model has depicted effective precision-recall values on the classification of two classes, namely, class 0 and class 1 on the test Leukemia dataset.

**Figure 5:**
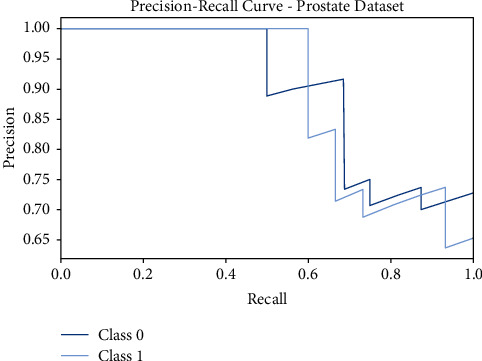
Precision-recall analysis of FSS-OANFIS technique under the Prostate dataset.

Next, Figure 5 shows the precision-recall curves offered by the FSS-OANFIS model on the test Prostate dataset. The figure revealed that the FSS-OANFIS technique has depicted effective precision-recall values on the classification of two classes, namely, class 0 and class 1 on the test Prostate dataset.

**Figure 6:**
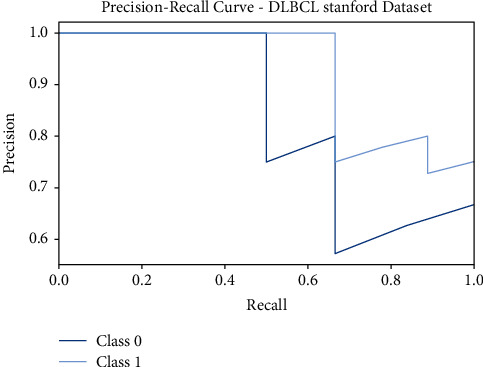
Precision-recall analysis of FSS-OANFIS technique under the DLBCL Stanford dataset.

Similarly, Figure 6 shows the precision-recall curves offered by the FSS-OANFIS system on the test DLBCL Stanford dataset. The figure exposed that the FSS-OANFIS model has depicted effective precision-recall values on the classification of two classes, namely, class 0 and class 1 on the test DLBL Stanford dataset.

**Figure 7:**
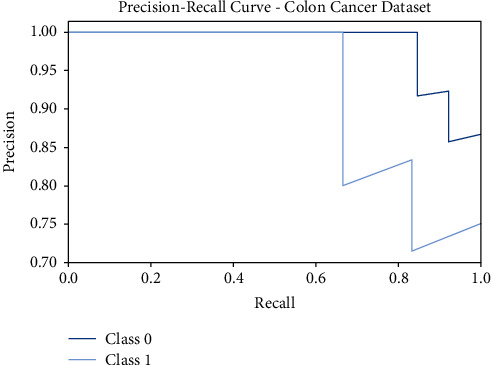
Precision-recall analysis of FSS-OANFIS technique under the Colon Cancer dataset.

Figure 7 shows the precision-recall curves offered by the FSS-OANFIS method on the test Colon Cancer dataset. The figure indicated that the FSS-OANFIS approach has depicted effective precision-recall values on the classification of two classes, namely, class 0 and class 1 on the test Colon Cancer dataset.

**Figure 8:**
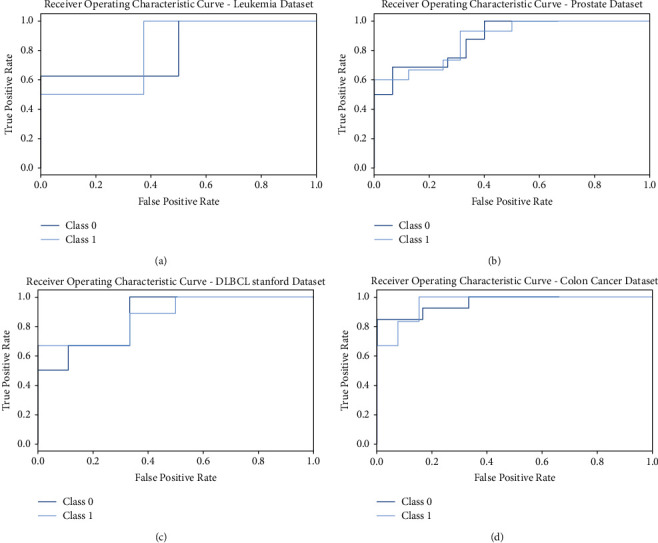
ROC analysis of FSS-OANFIS technique under different datasets.

A brief ROC investigation of the FSS-OANFIS model on the distinct four datasets is described in Figure 8. The results indicated that the FSS-OANFIS technique has exhibited its ability in categorizing two different classes such as class 0 and 1 on the test four datasets.

**Figure 9:**
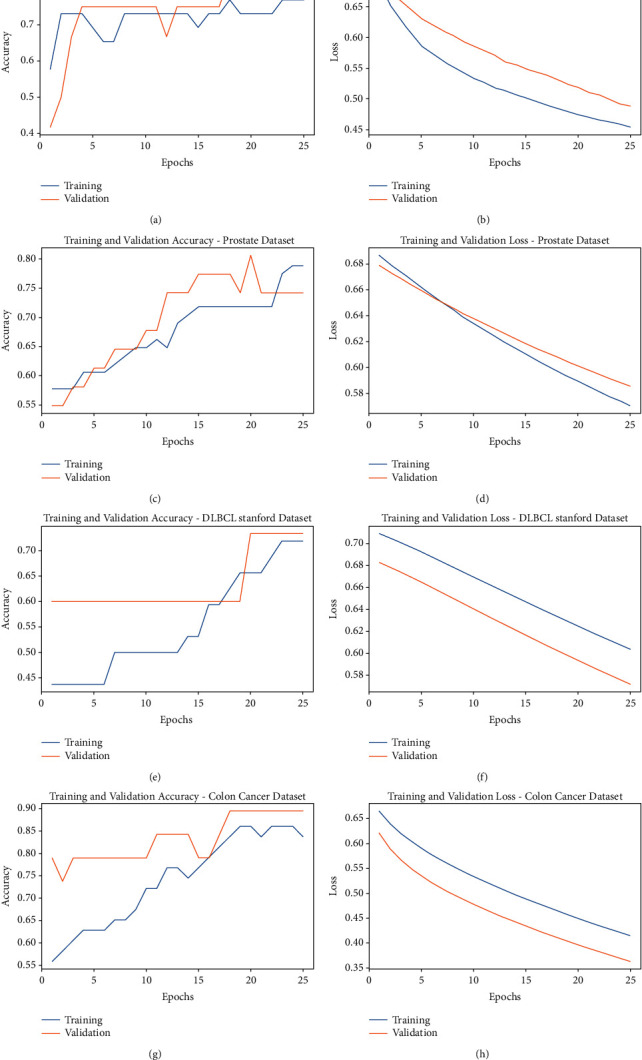
Accuracy and loss analysis of FSS-OANFIS technique under various datasets.

Figure 9 shows the accuracy and loss graph analysis of the ODBN-IDS technique under four datasets. The results show that the accuracy value tends to increase and the loss value tends to decrease with an increase in epoch count. It is also observed that the training loss is low and validation accuracy is high under four datasets.

### 4.2. Discussion

**Figure 10:**
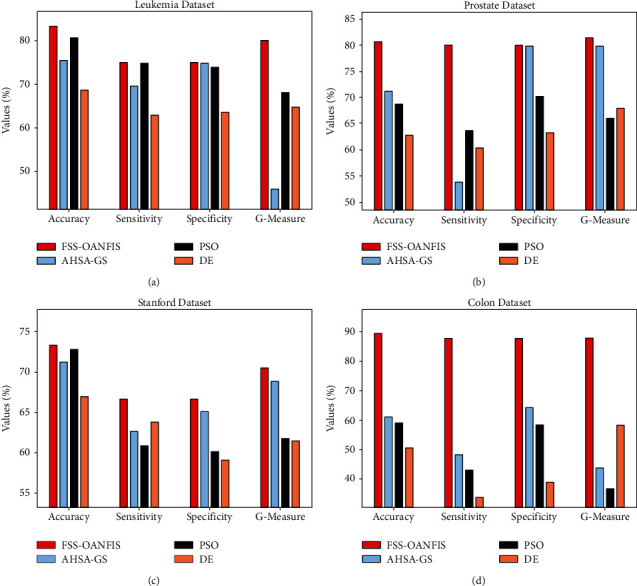
Comparative analysis of FSS-OANFIS technique with existing approaches.

Finally, a detailed comparative study of the FSS-OANFIS model with recent methods on distinct datasets is shown in Table 3 and Figure 10 [27]. The experimental results indicated that the FSS-OANFIS model has shown effectual outcomes under all datasets. For instance, with the Leukemia dataset, the DE and AHSA-GS models have depicted lower performance over the other methods. Though the PSO algorithm has resulted in slightly reasonable performance with accu_*y*_, sens_*y*_, spec_*y*_, and *G*_measure_ of 80.59%, 74.95%, 73.96%, and 68.07%, the FSS-OANFIS model has resulted in higher accu_*y*_, sens_*y*_, spec_*y*_, and *G*_measure_ of 83.33%, 75%, 75%, and 80.08%, respectively.

At the same time, with the Prostate dataset, the DE and AHSA-GS models have depicted lower performance over the other methods. Likewise, the PSO algorithm has resulted in somewhat reasonable performance with *accu*_*y*_, *sens*_*y*_, *spec*_*y*_, and *G*_measure_ of 68.78%, 63.63%, 70.15%, and 66.01% and the FSS-OANFIS methodology has resulted in superior accu_*y*_, sens_*y*_, spec_*y*_, and *G*_measure_ of 80.65%, 80%, 80%, and 81.37%, respectively. In addition, with the DLBCL Stanford dataset, the DE and AHSA-GS techniques have showcased lesser performance over the other methods. Though the PSO algorithm has resulted in slightly reasonable performance with accu_*y*_, sens_*y*_, spec_*y*_, and *G*_measure_ of 72.80%, 60.82%, 60.17%, and 61.74%, the FSS-OANFIS approach has resulted in higher accu_*y*_, sens_*y*_, spec_*y*_, and *G*_measure_ of 733.33%, 66.67%, 66.67%, and 70.47%, respectively.

Along with that, with the Colon Cancer dataset, the DE and AHSA-GS models have portrayed lower performance over the other methods. But, the PSO approach has resulted in slightly reasonable performance with *accu*_*y*_, *sens*_*y*_, *spec*_*y*_, and *G*_measure_ of 59%, 43.02%, 58.34%, and 36.66%, and the FSS-OANFIS system has resulted in superior accu_*y*_, sens_*y*_, spec_*y*_, and *G*_measure_ of 89.47%, 87.80%, 87.82%, and 87.82%, respectively. After examining the results and discussion, it is apparent that the FSS-OANFIS model has accomplished maximum performance in the microarray gene expression classification process.

## 5. Conclusion

In this study, a new FSS-OANFIS model has been developed for microarray gene expression data classification. The presented FSS-OANFIS model encompasses a series of processes, namely, data pre-processing, IGWO-FS-based election of features, ANFIS classification, and COA-based parameter optimization. The application of IGWO-FS and COA techniques helps in accomplishing enhanced microarray gene expression classification outcomes. For examining the enhanced outcomes of the FSS-OANFIS model, a wide range of simulations were performed on distinct datasets. The experimental results indicated that the FSS-OANFIS model has resulted in enhanced performance over the recent approaches. In future, the feature reduction and clustering approaches can be integrated to enhance gene expression classification outcomes.

## Figures and Tables

**Table 1:** Dataset details.

Dataset	Leukemia	Prostate	DLBCL Stanford	Colon Cancer
No. of genes	7129	12600	4026	2000
Class 0	27	52	24	40
Class 1	11	50	23	22
Total no. of samples	38	102	47	62

**Table 2:** Result analysis of FSS-OANFIS technique with different measures and datasets.

Class labels	Accuracy	Recall	Specificity	F-score	G-measure
Leukemia dataset					

Class 0	83.33	100.00	50.00	88.89	89.44

Class 1	83.33	50.00	100.00	66.67	70.71

Average	83.33	75.00	75.00	77.78	80.08

Prostate dataset					

Class 0	80.65	100.00	60.00	84.21	85.28

Class 1	80.65	60.00	100.00	75.00	77.46

Average	80.65	80.00	80.00	79.61	81.37

Stanford dataset					

Class 0	73.33	33.33	100.00	50.00	57.74

Class 1	73.33	100.00	33.33	81.82	83.21

Average	73.33	66.67	66.67	65.91	70.47

Colon dataset					

Class 0	89.47	92.31	83.33	92.31	92.31

Class 1	89.47	83.33	92.31	83.33	83.33

Average	89.47	87.82	87.82	87.82	87.82

**Table 3:** Comparative analysis of FSS-OANFIS technique with existing approaches.

Methods	Accuracy	Sensitivity	Specificity	G-measure
Leukemia dataset				

FSS-OANFIS	83.33	75.00	75.00	80.08

AHSA-GS	75.49	69.66	74.81	45.94

PSO algorithm	80.59	74.95	73.96	68.07

DE algorithm	68.67	63.01	63.62	64.80

Prostate dataset				

FSS-OANFIS	80.65	80.00	80.00	81.37

AHSA-GS	71.19	53.82	79.79	79.84

PSO algorithm	68.78	63.63	70.15	66.01

DE algorithm	62.77	60.37	63.22	67.94

Stanford dataset				

FSS-OANFIS	73.33	66.67	66.67	70.47

AHSA-GS	71.27	62.64	65.15	68.82

PSO algorithm	72.80	60.82	60.17	61.74

DE algorithm	66.93	63.80	59.16	61.48

Colon dataset				

FSS-OANFIS	89.47	87.82	87.82	87.82

AHSA-GS	61.02	48.07	64.04	43.62

PSO algorithm	59.00	43.02	58.34	36.66

DE algorithm	50.38	33.63	38.76	58.07

## Data Availability

Data are available and can be provided upon direct request to the corresponding author.
